# Supplementary material for: Single-pixel imaging with Morlet wavelet correlated random patterns
Source: Sci Rep. 2018 Jan 11;8:466. doi: 10.1038/s41598-017-18968-6 (PMC5765164; doi:10.1038/s41598-017-18968-6)
Supplement: Supplementary file 1 — Supplementary Information [file 41598_2017_18968_MOESM1_ESM.pdf]

Supplementary materials for Single-pixel imaging with Morlet wavelet correlated random patterns

Krzysztof M. Czajkowski, Anna Pastuszczak, and Rafal Kotynski

S1 Approximate decomposition of an image database into Morlet wavelets

In this section we explain our method of calculating the average decomposition of an image database into Morlet wavelets, which is presented in Fig. 2, and which is later used to select the Morlet wavelets for the sampling functions. We also discuss the relation of the decomposition into Fourier basis and Morlet wavelets.

A Morlet wavelet function $g_{\sigma,n_p,\theta}(x-x_0, y-y_0)$ is defined with 5 parameters (See Equation (2)), namely the size of Gaussian envelope σ, the number of peaks within the envelope n_p, orientation θ, as well as the location of the center of the wavelet (x_0, y_0). Our purpose was to determine which part of the feature space spanned by these parameters is needed to represent typical real-world images. Assuming no preference for the spatial location of the features (x_0, y_0) and their possible rotation θ, we focused only on two parameters (σ, n_p).

We were therefore interested in finding the most common range of values of (σ, n_p) appearing in an approximate decomposition of a set of images into Morlet wavelets. Following, our sampling functions $\Psi_{\sigma,n_p,\theta}(x, y)$ have been constructed as a convolution of white noise with Morlet wavelets $g_{\sigma,n_p,\theta}(x, y)$ defined by σ, and n_p selected randomly from the range of interest, and θ selected randomly from the range $[0, 2\pi[$. Sampling an image with these functions is equivalent to sampling the respective feature vector $X_{x_0,y_0,\sigma,n_p,\theta}$ (i.e. the representation of the image in the feature space $(x_0, y_0, \sigma, n_p, \theta)$) randomly with a uniform distribution over x_0, y_0, θ but with a nonuniform distribution over σ, n_p. These two parameters are taken randomly from the estimated region of interest.

S1.1 Calculation method

We should note that Morlet wavelets are in general not orthogonal, as well as that the expansion of an image into Morlet wavelets is not unique. Finding the most efficient expansion is computationally challenging, so we took a simplified approximate approach. Decomposition of a vector into a linear combination of non-orthogonal vectors such as Morlet wavelets is equivalent to solving a system of linear equations $Ax = b$, where columns of A are the Morlet wavelets, x are the unknown expansion coefficients and b is the decomposed vector. When the number of linearly independent Morlet wavelets in A equals the number of pixels in the image, the decomposition is exact and unique, though the choice of the wavelets from the continuous parameter space itself is not unique. As the inversion of a $512^2 \times 512^2$ matrix is not possible with limited computational resources, we decided to take a different approach. For each decomposition we selected a set of 10^4 wavelets and calculated

an approximate decomposition using pseudoinverse $x = A^{+}b$ (where the size of A is $512^2 \times 10^4$). We have calculated this expression 490 times (10 times with different matrix A for each of 49 images b from the image database). The average occurrences of (σ, n_p) in these decompositions is plotted in Fig. 2. In order to select the Morlet wavelets included in matrix A, we cropped the space down to region with relatively low spatial frequencies ($n_p < 15$) and reasonable Gaussian size (no larger Gaussian envelopes than the image were allowed). For each Morlet wavelet we selected random location (x_0, y_0) and orientation angle θ, while (σ, n_p) took discrete values from the predetermined region. We calculated the decomposition 10 times with different sets of (x_0, y_0, λ) for each image to average over feature location and orientation angle. Finally, Fig. 2. shows the mean value of the absolute values of the 490 decompositions projected onto (σ, n_p).

S1.2 Relation to the Fourier basis

Fourier basis functions are a special subset of Morlet wavelets with $\omega_x = \pi n_p \cos(\theta)/2\sigma$, $\omega_y = \pi n_p \sin(\theta)/2\sigma$, and $\sigma \to \infty$. An expansion of an image into this orthogonal subset of Morlet wavelets is unique, but may be less sparse than other possible expansions. Moreover, while sampling an image with Fourier basis provides the highest possible resolution in the frequency domain, other Morlet wavelets allow to achieve a trade-off between the sampling resolution in the frequency and spatial domains.

Usually, the spatial spectrum of real-world images is dominated by the low-frequency content. In the Morlet-wavelet representation this would correspond to small values of n_p and large values of σ. This is not very different from what we see in the decomposition presented in Fig. 2 in the main text, which is centered at small values of n_p. We have also verified that indeed, the Morlet-wavelet representation still corresponds to low-frequency Fourier representation (images reconstructed directly from the incomplete Morlet-wavelet representation still have the spatial spectrum dominated by low-frequency content). Though the optimal Morlet-wavelet decomposition does not have to be similar to the Fourier decomposition, as it is also sensitive to the typical range of feature sizes in images. The units used by us for σ roughly correspond to the percentage of the image width (we assumed that 3σ is equal to the size of the image equal to 512 or 256 pixels). The maximal values of σ are distributed in the range of $15\% - 40\%$ corresponding to the same range of feature sizes in the images.

S2 Comparison of the image reconstruction quality at various compression rates

In this section we show additional reconstruction results to better illustrate the properties of the proposed sampling method. Fig. 1 shows examples of numerical reconstructions obtained with the pseudoinverse of the measurement matrix for the three sampling schemes - with randomly selected Walsh-Hadamard and noiselet sampling as well as with the proposed Morlet-wavelet-based method. These results have been obtained for a 512×512 image sampled at the compression rate of 4% and can be compared to the CS results from Fig. 3 of the main paper. Neither Walsh-Hadamard nor noiselet functions enable clear reconstruction at this compression rate. Especially striking is the difference between the CS-based and the pseudoinverse-based reconstructions obtained with noiselet sampling functions. The CS-based reconstruction is very good, while the pseudoinverse-based one visually resembles white noise. The CS-based reconstruction depends on the incoherence between the measurement and compression matrices, and noiselets are known to have a small coherence with compression operators based on wavelet transforms. Therefore they work well with CS-based methods. On the other hand, the noiselet representation of typical images is usually rather uniform, which results in a poor reconstruction quality obtained with the pseudoinverse. At the same time, the pseudoinverse of the image sampled with Morlet-wavelet-based functions is very good.

In Fig. 2 we compare the CS and pseudoinverse based reconstructions from the optical measurements conducted at various compression rates (between 3% and 15% at the resolution of 256×256). Sampling is based on the binarized Morlet-wavelet-based random patterns. The image is clearly visible even when the compression ratio equals 3%, however the quality of pseudoinverse-based reconstruction becomes poor at a small compression ratio. This is accordance with the PSNR results shown in Fig. 7 of the paper.

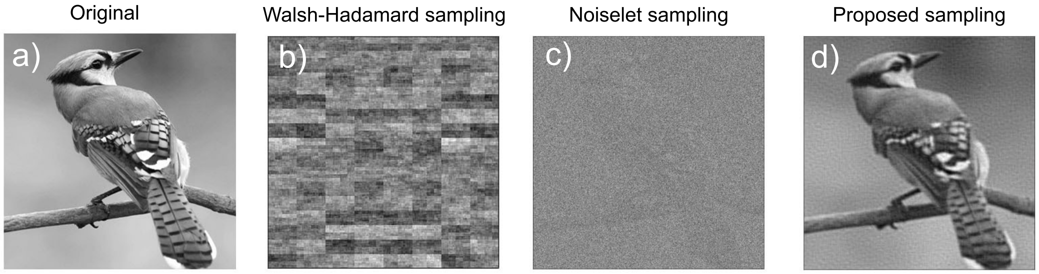

Figure 1: Pseudoinverse-based reconstructions of the 512×512 image measured in a simulation at the compression rate of 4% with various sampling protocols. a) original image; b) reconstruction from a compressive measurement, where sampling was based on randomly selected Walsh-Hadamard functions;c) reconstruction from a compressive measurement, where sampling was based on randomly selected discrete noiselet functions; d) reconstruction from a compressive measurement, where sampling was based on the proposed binarized Morlet wavelet-based random functions.

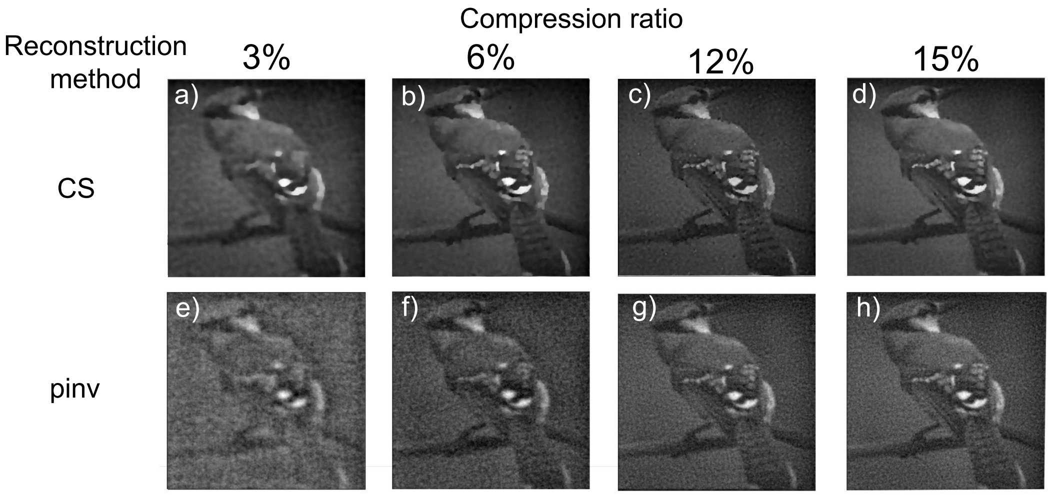

Figure 2: CS-based and pseudoinverse-based reconstructions obtained at the resolution of 256×256 at various compression rates from the optical measurement. Sampling was based on the proposed Morlet wavelet-based random functions.
